# Aza-Michael Mono-addition Using Acidic Alumina under Solventless Conditions

**DOI:** 10.3390/molecules21060815

**Published:** 2016-06-22

**Authors:** Giovanna Bosica, Roderick Abdilla

**Affiliations:** Department of Chemistry, University of Malta, Msida MSD 2080, Malta; roderick.abdilla.12@um.edu.mt

**Keywords:** Aza-Michael reactions, mono-addition, acidic alumina, solvent-free, primary amines

## Abstract

Aza-Michael reactions between primary aliphatic and aromatic amines and various Michael acceptors have been performed under environmentally-friendly solventless conditions using acidic alumina as a heterogeneous catalyst to selectively obtain the corresponding mono-adducts in high yields. Ethyl acrylate was the main acceptor used, although others such as acrylonitrile, methyl acrylate and acrylamide were also utilized successfully. Bi-functional amines also gave the mono-adducts in good to excellent yields. Such compounds can serve as intermediates for the synthesis of anti-cancer and antibiotic drugs.

## 1. Introduction

The aza-Michael addition involves the formation of a C-N bond between nitrogen donors and α,β-unsaturated compounds [1,2,3]. This reaction is particularly important in the production of antibiotics, anticancer agents and bioactive molecules such as β-amino acid oligomers that can mimic the biological activity of cationic α-helical antimicrobial peptides without getting broken down by the body [4,5].

**Scheme 1:**
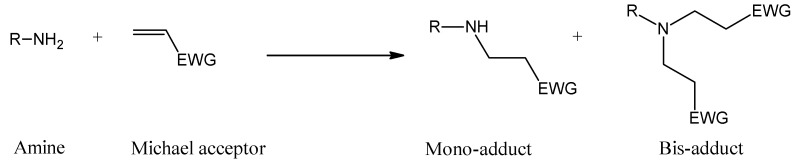
Aza-Michael mono- and bis-addition.

Primary amines react with Michael acceptors to form the corresponding mono-adduct, which can react further to give the bis-adduct (Scheme 1). Unfortunately it is quite difficult to selectively and separately obtain the mono-adduct and the bis-adduct from the same starting materials and in fact there has been little emphasis on this.

Originally the aza-Michael reaction was catalysed by harsh bases, which resulted in the formation of several side products [2]. With time came the advent of Lewis acid catalysts such as lanthanum trichloride [6], cerium (IV) ammonium nitrate (V) [7], zirconium (IV) chloride [8], samarium (III) triflate [9], and cadmium (II) chloride [10]. These catalysts present a lot of disadvantages: they are expensive, require harsh conditions and hazardous solvents, need relatively long reaction times, and they are homogeneous and hence difficult to separate and recycle. Ionic liquids, despite being homogeneous, have also became popular and provide good results, despite the fact that the procedures involving their reuse and recovery are always time-consuming, elaborate and costly [11].

Recently there has been an ideological shift towards green organic chemistry [12,13], not least in the aza-Michael reaction. As a result there has been the advent of heterogeneous catalysts such as silica-supported sulphuric (VI) acid [14], polymer-supported catalysts [15,16] metal organic frameworks [17], graphene oxide [18], Amberlyst-15 [19], and basic alumina [20]. In addition of microwave- and ultrasound-assisted reactions have been introduced [21]. Although most of these catalysts are recoverable, these studies have rarely focused on selective formation of mono-adducts from primary amines or they only focused on a few substrates. In some cases toxic solvents were still required and long reaction times were needed.

Continuing our efforts to explore heterogeneous catalysis in organic synthesis under green conditions [22] we have recently reported an environmentally-friendly procedure to efficiently obtain selectively mono- or bis- aza-Michael adducts using acidic alumina as heterogeneous catalyst [23].

Consequently, in continuation of previous studies performed by our research group on aza-Michael reactions, we here further explore the scope and efficiency of acidic alumina as a heterogeneous catalyst for aza-Michael additions [23,24]. We have widened the range of substrates in order to selectively form mono-adducts in solvent-free conditions under reflux.

## 2. Results and Discussion

Following our previously optimized procedure all reactions were performed by mixing the two starting materials in the presence of 0.2 g of acidic alumina per mmol of substrate whilst heating to reflux under solventless conditions [23]. The molar ratio of the Michael donor and acceptor was always kept at 1.5:1 and the aza-Michael adduct products were purified by column chromatography. We found that several Michael acceptors, different from methyl acrylate, as well as a variety of functionalised amines, together with a combination of both, were also effective under the developed procedure conditions, confirming the versatility and main advantages of this catalyst. In fact it does not require special preparation, it is cheap and easily available and it is used under neat conditions.

Ethyl acrylate (**2**) (Table 1) was the main Michael acceptor used to study the activity of the catalyst. Linear aliphatic primary amines (entries 1 and 2) provided good yields (78%–76%) albeit lower than those of cyclic ones, as expected according to their smaller steric hindrance. *c*-Pentylamine (entry 4) for example, produced the mono-adduct at 90% yield after heating for 3 h at 70–80 °C. Multi-functional amines such as allyl amine and propargyl amine (entries 5 and 6) gave good to excellent results, showing that negative inductive effects are short ranged and not very effective in decreasing the electron density on the nitrogen. However, 2-aminobutanol (entry 3) gave a lower yield because of the competing oxa-Michael addition and probably also because of product adsorption onto the catalyst. Meanwhile, primary aromatic amines (entries 8, 10 and 11) afforded the mono-adducts in excellent yields (89%–98%) in between 3 and 5 h, whereas a poor yield was only obtained when 2-aminothiazoline (entry 7) was used as a Michael donor, even after allowing the reaction to proceed for over 68 h at 90 °C. The end product was a thick yellow oil with a pungent smell similar to that of rotten eggs. A possible reason for the low yield could be the negative mesomeric effect which decreases the charge density on the nitrogen [3]. The good yields obtained for functionalised amines stimulated us to try them out with methyl acrylate (**5**) as Michael acceptor and very good results were once more obtained (Table 2).

Other Michael acceptors were then tested and tried out (Table 3). Excellent yields were obtained for acrylonitrile acceptor (83%–100%) and for acrylamide (90%–95%) despite the fact that for the latter acceptor slightly longer reaction times were needed (entries 8 and 9). Contrastingly, the yields obtained for the additions of *n*-alkylamines to methyl methacrylate (**12**), methyl *trans*-crotonate (**14**) and methyl *trans*-cinnamate (**16**) were slightly less impressive, even if the reaction time was increased (entries 10–14). These observations can be explained in terms of steric reasons. To further prove this, no bis-adduct was observed to be formed during the course of their reaction.

Finally other challenging Michael acceptors were tested. When β-nitrostyrene was used, the mono-adduct which was supposedly formed could not be characterized by ^1^H-NMR. This could be because the mono-adduct or the Michael acceptor itself were not stable. In fact, it is reported that β-nitrostyrene undergoes [2 + 2] cycloaddition in the presence of sunlight [25]. Even when the reaction was repeated in the absence of light, the product obtained was still not characterized by proton NMR because the crude was exposed to light during column chromatography. Moreover, the silica used in the column could itself have caused the product to decompose. Styrene yielded only very small traces with *n*-butylamine, whilst no product was formed with aniline. This confirmed that without the presence of electron withdrawing groups, the benzene ring by itself is not enough to decrease the electron density in the double bond.

The products of addition of primary *n*-alkyl amines to α,β-unsaturated aldehydes/ketones such as: *trans*-cinnamaldehyde, 2-hexenal, 2-heptenal, 2-cyclopentenone and 2-cyclohexenone could not be characterized. When column chromatography was performed, the eluted products which were obtained soon turned dark and very viscous. A probable explanation for this could be that these acceptors were forming α,β-unsaturated imines instead of the mono-adducts. These are reportedly very unstable and can oligomerize easily [26].

## 3. Materials and Methods

### 3.1. General Information

All commercially available chemicals were purchased from Aldrich (St. Louis, MO, USA) and used without further purification. Acidic alumina (grain size: 0.05–0.2 mm, 70–290 mesh ASTM, pH 4.5, activity degree 1, Scharlau, Barcelona, Spain) was used without further activation. IR spectra were recorded on a IRAffinity-1 FTIR spectrometer (Shimadzu, Kyoto, Japan) calibrated against a 1602 cm^−1^ polystyrene absorbance spectrum. Samples were analysed as a thin film or in a Nujol™ mull between sodium chloride plates. The ^1^H and ^13^C-NMR spectra were recorded on an Avance III HD^®^ NMR spectrometer (Bruker, Coventry, England), equipped with an Ascend 500 11.75 Tesla Superconducting Magnet, operating at 500.13 MHz for ^1^H and 125.76 MHz for ^13^C, and a Multinuclear 5 mm PABBO Probe (Bruker, Coventry, England). Samples were dissolved in deuterated chloroform (with TMS). For a few products NMR analysis was performed using a Bruker AM250 NMR spectrometer fitted with a dual probe at frequencies of 250 MHz for ^1^H-NMR and 62.9 MHz for ^13^C-NMR. Processing was carried out using an Aspect 3000 computer having 16 K and 64 K complex points for ^1^H and ^13^C-NMR respectively. Mass spectra were performed using a ACQUITY^®^ TQD system (Waters^®^,En Yvelines Cedex, France) with a tandem quadrupole mass spectrometer after dissolving the sample in methanol. Reactions were monitored using TLC and GC on a Shimadzu GC-2010 *plus* gas chromatograph equipped with a flame ionisation detector and HiCap 5 GC column with dimensions of 0.32 mm (internal diameter) × 30 m (length) × 0.25 mm (film thickness), using nitrogen as carrier gas.

### 3.2. Procedure for Preparation of Mono-Adducts

The amine (7.5 mmol) and the Michael acceptor (5 mmol) in a molar ratio of 1.5:1 were refluxed with stirring in the presence of acidic alumina (1 g, 200 mol%). Heating was performed using an oil bath and the reaction was followed by TLC and GC until completion. The reaction was then allowed to cool down to room temperature and filtered through a filter paper. The catalyst was rinsed with ethyl acetate/hexane and then concentrated by rotary evaporation. The crude reaction mixture was purified using a silica-filled chromatographic column using hexane/ethyl acetate as eluents. Usually, for aliphatic amines, the mono-adduct was eluted using 7:3, 6:4 or 5:5 hexane/ethyl acetate whilst for aromatic ones the solvent mixture used was 8:2 hexane/ethyl acetate. The yields of the purified products were recorded and then IR and NMR spectroscopy and MS spectrometry were performed.

### 3.3. Product Identification

*Ethyl 3-(butylamino)propanoate* (**3a**) [26,27]. Yellow oil. IR (neat, cm^−1^): ν = 3323, 2958, 2931, 2860, 1724, 1463, 1373, 1348, 1184, 1126, 1030, 852, 787. ^1^H-NMR (CDCl_3_, 500 MHz): δ (ppm) 4.14 (q, *J* = 7.2 Hz, 2H), 2.88 (t, *J* = 6.5, 2H), 2.61 (t, *J* = 7.2 Hz, 2H), 2.51 (t, *J* = 6.6 Hz, 2H), 1.50–1.39 (m, 2H), 1.38–1.33(m, 2H), 1.26 (t, *J* = 7.2 Hz, 3H), 0.91 (t, *J* = 7.4, 3H).

*Ethyl 3-(hexylamino)propanoate* (**3b**) [28]. Yellow oil. IR (neat, cm^−1^): ν = 3323, 2957, 2927, 2857, 1732, 1456, 1373, 1180, 1126, 1030, 787. ^1^H-NMR (CDCl_3_, 500 MHz): δ (ppm) 4.14 (q, *J* = 7.2 Hz, 2H), 2.88 (t, *J* = 6.5 Hz, 2H), 2.60 (t, *J* = 7.2 Hz, 2H), 2.51 (t, *J* = 6.6 Hz, 2H), 1.47 (t, *J* = 7.1 Hz, 3H), 1.33–1.25 (m, 8H), 0.88 (t, *J* = 6.9 Hz, 3H). ^13^C-NMR (CDCl_3_, 126 MHz): δ (ppm) 172.81, 60.32, 49.82, 45.09, 34.78, 31.74, 30.01, 26.98, 22.58, 14.18, 14.00. MS (ES+) *m*/*z* (%) = 202 [MH^+^] (20), 114 (100), 44 (48).

*Ethyl 3-(1-hydroxybutan-2-ylamino)propanoate* (**3c**). Very thick yellow oil. IR (neat, cm^−1^): ν = 3362, 3316, 2965, 2934, 2876, 1724, 1558, 1454, 1373, 1348, 1313, 1249, 1250, 1188, 1146, 1096, 1049, 1032, 794. ^1^H-NMR (CDCl_3_, 500 MHz): δ (ppm) 4.16 (q, *J* = 7.2 Hz, 2H), 3.61 (dd, *J* = 10.7, 4.0 Hz, 1H), 3.28 (dd, *J* = 10.70, 6.8 Hz, 1H), 3.02–2.97 (m, 1H), 2.83–2.79 (m, 1H), 2.55–2.53 (m, 1H), 2.49 (t, *J* = 6.4 Hz, 2H), 1.54–1.41 (m, 2H), 1.27 (t, *J* = 7.2 Hz, 3H), 0.93 (t, *J* = 7.5 Hz, 3H). ^13^C-NMR (CDCl_3_, 126 MHz): δ (ppm) 172.83, 62.64, 60.52, 60.23, 42.00, 35.09, 24.20, 14.16, 10.34. MS (ES+) *m*/*z* (%) = 190 [MH^+^] (22), 102 (100), 30 (8).

*Ethyl 3-(cyclopentylamino)propanoate* (**3d**) [29]. Yellow oil. IR (neat, cm^−1^): ν = 3323, 2955, 2868, 1728, 1465, 1373, 1350, 1242, 1184, 1165, 1047, 1030, 854, 785. ^1^H-NMR (CDCl_3_, 500 MHz): δ (ppm) 4.14 (q, *J* = 7.2 Hz, 2H), 3.07 (quin, *J* = 6.8 Hz, 1H), 2.86 (t, *J* = 6.6 Hz, 2H), 2.51 (t, *J* = 6.6 Hz, 2H), 1.88–1.81 (m, 2H), 1.73–1.64 (m, 3H), 1.56–1.48 (m, 2H), 1.38–1.29 (m, 2H), 1.26 (t, *J* = 7.2 Hz, 3H). ^13^C-NMR (CDCl_3_, 126 MHz): δ (ppm) 176.34, 52.80, 51.55, 49.80, 39.94, 29.69, 22.54, 13.99. MS (ES+) *m*/*z* (%) = 186 [MH^+^] (30), 98 (100), 30 (26).

*Ethyl 3-(prop-2-en-1-ylamino)propanoate* (**3e**) [30]. Light-yellow oil. IR (neat, cm^−1^): ν = 3323, 3076, 2980, 1736, 1643, 1558, 1463, 1456, 1373, 1254, 1242, 1184, 1115, 1030, 997, 918, 790. ^1^H-NMR (CDCl_3_, 500 MHz): δ(ppm) 5.91–5.83 (m, 1H), 5.17 (dq, *J* = 17.1, 1.6 Hz, 1H), 5.08 (dq, *J* = 10.3, 1.4 Hz, 1H), 4.12 (q, *J* = 7.2 Hz, 2H), 3.25 (t, *J* = 6.1 Hz, 2H), 2.87 (t, *J* = 6.5 Hz, 2H), 2.50 (t, *J* = 6.5 Hz, 2H), 1.77 (s, NH, 1H), 1.24 (t, *J* = 7.2 Hz, 3H). ^13^C-NMR (CDCl_3_, 126 MHz): δ (ppm) 172.67, 136.40, 116.11, 60.39, 52.11, 44.31, 34.62, 14.16.

*Ethyl 3-(propargylamino)propanoate* (**3f**)*.* Dark-yellow oil. IR (neat, cm^−1^): ν = 3418, 3391, 3291, 2982, 2935, 2909, 2851, 1724, 1466, 1459, 1373, 1258, 1184, 1119, 1096, 1030, 910, 856, 756. ^1^H-NMR (CDCl_3_, 500 MHz): δ (ppm) 4.15 (q, *J* = 7.2 Hz, 3H), 3.44 (d, *J* = 2.5 Hz, 1H), 2.97 (t, *J* = 6.5 Hz, 2H), 2.52 (t, *J* = 6.5 Hz, 2H), 2.20 (t, *J* = 2.4 Hz, 1H), 1.26 (t, *J* = 7.1 Hz, 3H). ^13^C-NMR (CDCl_3_, 126 MHz): δ (ppm) 172.58, 81.88, 71.50, 60.49, 60.39, 43.92, 38.10, 34.56. MS (ES+) *m*/*z* (%) = 156 [MH^+^] (46), 88 (13), 68 (100).

*Ethyl 3-(4,5-dihydro-1,3-thiazol-2-ylamino)propanoate* (**3g**) [31]. Yellow oil. IR (neat, cm^−1^): ν = 3395, 3051, 2955, 2858, 1728, 1651, 1612, 1558, 1504, 1447, 1416, 1373, 1354, 1308, 1277, 1238, 1169, 1115, 1042, 984, 941, 918, 928, 733, 698. ^1^H-NMR (CDCl_3_, 500 MHz): δ (ppm) 4.10 (q, *J* = 8.1 Hz, 2H), 3.75–3.61 (m, 2H), 3.61–3.45 (m, 2H). 3.22–3.14 (m, 2H), 2.71–2.59 (m, 2H), 1.24 (t, *J* = 8.3 Hz, 3H).

*Ethyl 3-(phenylamino)propanoate* (**3h**) [32]. Orange oil. IR (neat, cm^−1^): ν = 3401, 3053, 3022, 2980, 2933, 2904, 2870, 1736, 1720, 1604, 1558, 1506, 1375, 1317, 1251, 1180, 1114, 1099, 1047, 1028, 869, 858, 750, 692. ^1^H-NMR (CDCl_3_, 500 MHz): δ (ppm) 7.18 (t, *J* = 7.4 Hz, 2H), 6.72 (t, *J* = 6.4 Hz, 1H), 6.62 (d, *J* = 8.7 Hz, 2H), 4.14 (q, *J* = 7.2 Hz, 2H), 4.02 (broad s, 1H), 3.45 (t, *J* = 6.4 Hz, 2H), 2.61 (t, *J* = 6.4 Hz, 2H), 1.26 (t, *J* = 7.2 Hz, 3H).

*Ethyl 3-(benzylamino)propanoate* (**3i**) [30]. Yellow oil. IR (neat, cm^−1^): ν = 3325, 3086, 3062, 3028, 2981, 2904, 2835, 1732, 1496, 1454, 1373, 1350, 1180, 1119, 1095, 1029, 737, 698. ^1^H-NMR (CDCl_3_, 500 MHz): δ (ppm) 7.31 (d, *J* = 4.7 Hz, 4H), 7.28–7.19 (m, 1H), 4.14 (q, *J* = 7.1 Hz, 2H), 3.80 (s, 2H), 2.90 (t, *J* = 6.5 Hz, 2H), 2.53 (t, *J* = 6.5 Hz, 2H), 1.25 (t, *J* = 7.2 Hz, 3H).

*Ethyl 3-(4-ethylphenylamino)propanoate* (**3j**) [33]. Dark-brown oil. IR (neat, cm^−1^): ν = 3395, 3101 2963, 2932, 2870, 1732, 1616, 1520, 1473, 1458, 1396, 1373, 1315, 1242, 1180, 1126, 1095, 1045, 1026, 822. ^1^H-NMR (CDCl_3_, 500 MHz): δ (ppm) 7.00 (d, *J* = 8.5 Hz, 2H), 6.56 (d, *J* = 8.5 Hz, 2H), 4.14 (q, *J* = 7.1 Hz, 2H), 3.89 (broad s, 1H), 3.42 (t, *J* = 6.4 Hz, 2H), 2.59 (t, *J* = 6.4 Hz, 2H), 2.52 (q, *J* = 7.6 Hz, 2H), 1.25 (t, *J* = 7.2 Hz, 3H), 1.17 (t, *J* = 7.6 Hz, 3H). ^13^C-NMR (CDCl_3_, 126 MHz): δ (ppm) 172.47, 145.58, 133.64, 128.63, 113.28, 60.59, 39.83, 34.04, 27.93, 15.95, 14.21. MS (ES+) *m*/*z* (%) = 222 [MH^+^] (10), 134 (100), 119 (1).

*Ethyl 3-(4-methoxyphenylamino)propanoate* (**3k**) [34]. Dark-brown oil. IR (neat, cm^−1^): ν = 3383, 3237, 3067, 2986, 2955, 2940, 2909, 2835, 1724, 1627, 1513, 1465, 1458, 1442, 1373, 1296, 1238, 1180, 1119, 1092, 1034, 826, 760, 725. ^1^H-NMR (CDCl_3_, 500 MHz): δ (ppm) 6.77 (d, *J* = 9.0 Hz, 2H), 6.60 (d, *J* = 9.0 Hz, 2H), 4.14 (q, *J* = 7.1 Hz, 2H) 3.73 (s, 3H), 3.39 (t, *J* = 6.4 Hz, 2H), 2.58 (t, *J* = 6.4 Hz, 2H), 1.25 (t, *J* = 7.2 Hz, 3H).

*Ethyl 3-(isobutylamino)propanoate* (**3l**) [35]. Yellow oil. IR (neat, cm^−1^): υ = 2964, 2927, 2875, 2247, 1454, 1377, 698. ^1^H-NMR (CDCl_3_, 250 MHz): δ (ppm) 0.91 (t, *J* = 7.33 Hz, 3H), 1.05 (d, *J* = 6.1 Hz, 3H), 1.27–1.57 (m, 1H), 2.05 (s, 1H), 2.51 (t, *J* = 6.7 Hz, 1H), 2.61 (sx, *J* = 6.7 Hz, 1H), 2.85-3.05 (m, 1H), 3.48 (q, *J* = 6.7 Hz, 1H), 4.55 (q, *J* = 6.7 Hz, 1H). ^13^C-NMR (CDCl_3_, 62.9 MHz): δ (ppm) 10.1, 19.1, 19.8, 29.5, 42.5, 53.9, 118.8.

*Methyl 3-(prop-2-en-1-yl)propanoate* (**6e**) [36]. Light-yellow oil. IR (neat, cm^−1^): ν = 3323, 3076, 2953, 1736, 1728, 1643, 1558, 1456, 1436, 1364, 1238, 1196, 1177, 920, 854, 790. ^1^H-NMR (CDCl_3_, 500 MHz): δ(ppm) 5.91–5.83 (m, 1H), 5.18 (dq, *J* = 17.2, 1.7 Hz, 1H), 5.10 (dq, *J* = 10.3, 1.6 Hz, 1H), 3.69 (s, 3H), 3.26 (dt, *J* = 3.0, 1.4 Hz, 2H), 2.89 (t, *J* = 6.5 Hz, 2H), 2.53 (t, *J* = 6.5 Hz, 2H). ^13^C-NMR (CDCl_3_, 126 MHz): δ(ppm) 173.19, 136.51, 116.11, 52.19, 51.62, 44.38, 34.51.

*Methyl 3-(prop-2-yn-1-ylamino)propanoate* (**6f**). Dark-orange oil. IR (neat, cm^−1^): ν = 3291, 2982, 2954, 2928, 2851, 2098, 1732, 1458, 1438, 1373, 1246, 1177, 1119, 1045, 1018, 910, 844, 756. ^1^H-NMR (CDCl_3_, 500 MHz): δ (ppm) 3.70 (s, 3H), 3.44 (d, *J* = 2.4 Hz, 2H), 2.98 (t, *J* = 6.5 Hz, 2H), 2.54 (t, *J* = 6.5 Hz, 2H), 2.22 (t, *J* = 2.4 Hz, 1H). ^13^C-NMR (CDCl_3_, 126 MHz): δ (ppm) 172.56, 81.88, 71.51, 60.49, 48.99, 43.92, 38.10, 34.56, 14.21. MS (ES+) *m*/*z* (%) = 142 [MH^+^] (10), 75 (13), 68 (100).

*Methyl 3-(4-methoxybenzylamino)propanoate* (**6m**) [27]. Yellow oil. IR (neat, cm^−1^): ν = 3421, 3067, 2997, 2951, 2909, 2835, 1732, 1612, 1585, 1512, 1458, 1439, 1416, 1362, 1300, 1246, 1172, 1107, 1034, 818, 775, 756, 702. ^1^H-NMR (CDCl_3_, 500 MHz): δ (ppm) 7.21 (d, *J* = 8.7 Hz, 2H), 6.80 (d, *J* = 8.7 Hz, 2H), 3.78 (s, 3H), 3.72 (s, 2H), 3.66 (s, 3H), 2.87 (t, *J* = 6.5 Hz, 2H), 2.52 (t, *J* = 6.5 Hz, 2H).

*3-(Hexylamino)propanenitrile* (**9b**) [37]. Pale yellow oil. IR (neat, cm^−1^): υ = 3313, 2954, 2990, 2856, 2247, 1465, 1377, 1128. 1H-NMR (CDCl_3_, 250 MHz): δ (ppm) 0.85-0.95 (m, 3H), 1.20–1.40 (m, 6H), 1.40–1.55 (m, 3H), 2.53 (t, *J* = 6.3 Hz, 2H), 2.62 (t, *J* = 6.7 Hz, 2H), 2.92 (t, *J* = 6.72 Hz, 2H). ^13^C-NMR (CDCl_3_, 62.9 MHz): δ (ppm) 14.0, 18.7, 22.6, 26.9, 30.0, 31.75, 45.1, 49.3, 118.8.

*3-(Cyclopentylamino)propanenitrile* (**9d**) [38]. Yellow oil. IR (neat, cm^−1^): ν = 3310, 2955, 2866, 2245, 1473, 1458, 1419, 1373, 1350, 1246, 1123, 1045, 875, 771. ^1^H-NMR (CDCl_3_, 500 MHz): δ (ppm) 3.09 (q, *J* = 6.7 Hz, 1H); 2.89 (t, *J* = 6.7 Hz, 2H), 2.50 (t, *J* = 6.6 Hz, 2H), 1.82–1.79 (m, 2H), 1.72–1.64 (m, 2H), 1.57–1.48 (m, 2H), 1.33–1.27 (m, 2H).

*3-(Prop-2-yn-1-ylamino)propanenitrile* (**9f**) [39]. Yellow oil. IR (neat, cm^−1^): ν = 3287, 2920, 2859, 2249, 2102, 1732, 1670, 1654, 1627, 1458, 1419, 1373, 1331, 1246, 1123, 1045, 910, 763, 656. ^1^H-NMR (CDCl_3_, 500 MHz): δ (ppm) 3.47 (d, *J* = 2.5 Hz, 2H), 3.00 (t, *J* = 6.7 Hz, 2H), 2.53 (t, *J* = 6.6 Hz, 2H), 2.40 (t, *J* = 2.5 Hz, 1H). MS (ES+) *m*/*z* (%) = 109 [MH^+^] (74), 68 (100), 39 (2).

*3-(Phenylamino)propanenitrile* (**9h**) [40]. Thick brown oil. IR (neat, cm^−1^): ν = 3410, 3363, 3217, 3036, 3013, 2928, 2249, 1620, 1605, 1507, 1496, 1465, 1419, 1312, 1269, 1176, 1119, 1026, 995, 880, 752, 694. ^1^H-NMR (CDCl_3_, 500 MHz): δ (ppm) 7.21–7.18 (m, 2H), 6.78–6.72 (m, 1H), 6.67 (dd, *J* = 8.5, 1.1 Hz, 2H), 3.63 (broad s, 1H), 3.52 (q, *J* = 6.5 Hz**,** 2H), 2.62 (t, *J* = 6.6 Hz, 2H).

*3-((Pyridin-2-ylmethyl)amino)propanenitrile* (**9n**) [41]. Orange oil. IR (neat, cm^−1^): υ = 3307, 3174, 2927, 2852, 2247, 1593, 1471, 1435, 1126, 997, 761. ^1^H-NMR (CDCl_3_, 250 MHz): δ(ppm) 2.56 (t, *J* = 6.71 Hz, 2H), 2.99 (t, *J* = 6.71 Hz, 2H), 3.99 (s, 2H), 7.19 (dd, *J* = 4.88, 7.32 Hz, 1H), 7.32 (d, *J* =7.33 Hz, 1H), 7.67 (td, *J* = 1.83, 7.33, 7.33 Hz, 1H), 8.53–8.60 (m, 1H). ^13^C-NMR (CDCl_3_, 62.9 MHz): δ (ppm)18.8, 44.7, 54.3, 118.7, 122.3, 122.4, 136.7, 149.3, 158.8.

*3-(4-Methoxybenzylamino)propanenitrile* (**9o**). Yellow oil. IR (neat, cm^−1^): ν = 3337, 3062, 3001, 2935, 2909, 2835, 1732, 1612, 1585, 1512, 1465, 1458, 1420, 1300, 1177, 1034, 1111, 1033, 817, 772, 756, 702. ^1^H-NMR (CDCl_3_, 500 MHz): δ (ppm) 7.22 (d, *J* = 8.7 Hz, 2H), 6.86 (d, *J* = 8.7 Hz, 2H), 3.79 (s, 3H), 3.76 (s, 2H), 2.91 (t, *J* = 6.6 Hz, 2H), 2.49 (t, *J* = 6.7 Hz, 2H). ^13^C-NMR (126 MHz, CDCl_3_): δ (ppm) 158.87, 131.60, 129.26, 118.76, 55.30, 52.58, 44.25, 18.77. MS (ES+) *m*/*z* (%) = 121 (100), 77 (43). (70), 69 (57) 71 (100), 30 (66).

*3-Isopropylamino)propanenitrile* (**9p**) [28]. Yellow oil. IR (neat, cm^−1^): ν = 3394, 3310. 2967, 2932, 2870, 2249, 1732, 1654, 1474, 1450, 1420, 1377, 1327, 1246, 1177, 1130, 1092, 1045, 848, 756. ^1^H-NMR (CDCl_3_, 500 MHz): δ (ppm) 2.93 (t, *J* = 6.7 Hz, 2H), 2.86 (sep, *J* = 6.3 Hz, 1H), 2.51 (t, *J* = 6.7 Hz, 2H), 1.08 (d, *J* = 6.3 Hz, 6H).

*3-(Butylamino)propanamide* (**11a**) [42]. Very thick colourless oil. IR (neat, cm^−1^): ν = 2986, 2940, 2909, 1739, 1446, 1373, 1242, 1049, 937, 848. ^1^H-NMR (CDCl_3_, 500 MHz): δ (ppm) 5.38 (broad s, 2H), 2.88 (t, *J* = 5.9 Hz, 2H), 2.63 (t, *J* = 7.1 Hz, 2H), 2.38 (t, *J* = 5.9 Hz, 2H), 1.51–1.45 (m, 2H), 1.36 (m, 2H), 0.92 (t, *J* = 7.3Hz, 3H).

*3-(Phenylamino)propanamide* (**11h**). Very thick light-yellow oil. IR (neat, cm^−1^): ν = 3341, 3194, 3045, 3012, 2963, 2862, 2245, 1664, 1645, 1614, 1508, 1423, 1320, 1265, 1180, 1118, 991, 910, 875, 810, 733, 694. ^1^H-NMR (CDCl_3_, 500 MHz): δ (ppm) 7.22 (t, *J* = 7.4 Hz, 2H), 6.76 (t, *J* = 7.4 Hz, 1H), 6.67 (d, *J* = 8.2 Hz, 2H), 5.35 (broad s, 2H), 3.96 (broad s, 1H), 3.51 (t, *J* = 6.0 Hz, 2H), 2.56 (t, *J* = 6.0 Hz, 2H). ^13^C-NMR (126 MHz, CDCl_3_): δ (ppm) 175.63, 49.15, 45.47, 35.35, 20.39, 13.90. MS (ES+) *m*/*z* (%) = 165 [MH^+^] (11), 106 (100), 77 (14).

*Methyl 3-(butylamino)-2-methylpropanoate* (**13a**) [26]. Yellow oil. IR (neat, cm^−1^): ν = 3327, 2957, 2932, 2874, 2860, 2821, 1740, 1558, 1463, 1454, 1435, 1377, 1361, 1255, 1196, 1199, 1177, 1165, 1138, 987, 833, 758. ^1^H-NMR (CDCl_3_, 500 MHz): δ (ppm) 3.69 (s, 3H), 2.88 (dd, *J* = 11.7, 7.9 Hz, 1H), 2.69–2.55 (m, 4H), 1.50–1.42 (m, 3H), 1.37–1.29 (m, 2H); 1.17 (d, *J* = 7.0 Hz, 3H), 0.91 (t, *J* = 7.4 Hz, 3H). ^13^C-NMR (CDCl_3_, 126 MHz): δ (ppm) 172.84, 62.64, 60.52, 60.23, 42.00, 35.10, 24.21, 14.17, 10.34. MS (ES+) *m*/*z* (%) = 174 [MH^+^] (30), 86 (100), 57 (2), 44 (16).

*Methyl 3-(hexylamino)-2-methylpropanoate* (**13b**) [43]. Yellow oil. IR (neat, cm^−1^): ν = 3327, 2955, 2928, 2872, 2857, 1732, 1558, 1463, 1456, 1435, 1377, 1361, 1259, 1201, 1175, 1138, 989, 893, 833, 761, 727. ^1^H-NMR (CDCl_3_, 500 MHz): δ (ppm) 3.69 (s, 3H), 2.88 (dd, *J* = 11.5, 7.8 Hz, 1H), 2.70–2.59 (m, 4H), 1.50–1.42 (m, 3H), 1.37–1.29 (m, 6H); 1.16 (d, *J* = 6.9 Hz, 3H), 0.88 (t, *J* = 7.1 Hz, 3H). MS (ES+) *m*/*z* (%) = 202.10 [MH^+^] (22), 114 (100), 44 (46).

*Methyl 3-(pentylamino)-2-methylpropanoate* (**13q**) [43]. Yellow oil. IR (neat, cm^−1^): ν = 3327, 2955, 2930, 2873, 2859, 1732, 1558, 1463, 1456, 1435, 1379, 1361, 1257, 1196, 1177, 1138, 1060, 989, 833, 750. ^1^H-NMR (CDCl_3_, 500 MHz): δ (ppm) 3.69 (s, 3H), 2.90 (dd, *J* = 11.6, 7.8 Hz, 1H), 2.74–2.52 (m, 4H), 1.50–1.42 (m, 2H), 1.34–1.30 (m, 5H), 1.19 (d, *J* = 7.0 Hz, 3H), 0.91 (t, *J* = 7.4 Hz, 3H). MS (ES+) *m*/*z* (%) = 210 [MH^+^] (100), 101 (20), 73 (7).

*Methyl 3-(butylamino)-3-methylpropanoate* (**15a**) [44]. Yellow oil. IR (neat, cm^−1^): ν = 3390, 2958, 2931, 2874, 1732, 1458, 1439, 1377, 1304, 1250, 1196, 1180, 1096, 1053, 1010, 879, 756, 710. ^1^H-NMR (CDCl_3_, 500 MHz): δ (ppm) 3.66 (s, 3H), 3.14–3.01 (m, 1H), 2.68–2.59 (m, 1H), 2.59–2.51 (m, 1H), 2.47 (dd, *J* = 15.3, 6.8 Hz, 1H), 2.32 (dd, *J* = 15.3, 6.1 Hz, 1H), 1.48–1.40 (m, 2H), 1.38–1.32 (m, 2H), 1.10 (d, *J* = 6.4 Hz, 3H), 0.90 (t, *J* = 7.3 Hz, 3H).

*Methyl 3-(butylamino)-3-phenylpropanoate* (**17a**) [45]. Thick light-yellow oil. IR (neat, cm^−1^): ν = 3395, 3063, 3028, 2954, 2932, 2862, 1716, 1663, 1635, 1578, 1543, 1496, 1450, 1435, 1373, 1330, 1315, 1277, 1242, 1204, 1173, 1045, 980, 864, 768, 702. ^1^H-NMR (CDCl_3_, 500 MHz): δ (ppm) 7.52–7.50 (m, 1H), 7.40–7.32 (m, 3H), 7.28–7.25 (m, 1H), 3.66 (s, 3H), 3.45–3.35 (m, 1H), 2.72 (dd, *J* = 15.6, 8.6 Hz, 1H), 2.63 (dd, *J* = 15.6, 5.4 Hz, 1H), 2.48–2.39 (m, 2H), 1.61–1.55 (m, 2H), 1.50–1.47 (m, 2H), 0.88 (t, *J* = 7.2 Hz, 3H). MS (ES+) *m*/*z* (%) = 221 [MH^+^] (7), 82 (100), 57 (53).

## 4. Conclusions

This study expanded the scope of our previously reported protocol for the synthesis of aza-Michael mono-adducts and confirmed the significant advantage of this method in producing the mono-adducts with high selectivity. Acidic alumina has shown to be a suitable catalyst to selectively obtain the mono-adducts in aza-Michael reactions with the additional advantage of the solvent-free heterogeneous conditions. A wide range of aliphatic/aromatic primary amines and Michael acceptor combinations have been tested successfully, while preserving several other functionalities.

All reactions were performed under green, heterogeneous and solventless conditions in the presence of 0.2 g of acidic alumina per mmol of substrate. Aliphatic amines gave good to excellent yields with the highest (100%) obtained in the addition of 4-methoxybenzylamine to acrylonitrile. Interestingly, cyclic amines provided better results than linear ones, whilst aromatic amines formed only the mono-adducts in excellent yields, with the highest yield of 98% in the reaction between ethyl acrylate and 4-methoxyaniline, and no trace of bis-addition product. Methyl methacrylate and methyl *trans*-cinnamate provided slightly lower yields due to steric hindrance. Bifunctional amines reacted successfully, the highest yield being that of 93% obtained for addition of propargylamine to ethyl acrylate.

## Figures and Tables

**Table 1:** Mono-addition of various primary amines **1a**–**l** to ethyl acrylate (**2**). 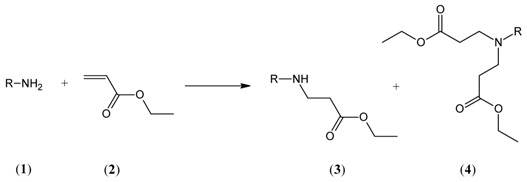

Entry	Amine (1)	Yield ^a^ 3:4 (%)	Temperature (°C)	Time (h) ^b^
1	*n*-C_4_H_9_NH_2_ (**1a**)	78:3	75	3
2	*n*-C_6_H_13_NH_2_ (**1b**)	76:4	70	3
3	NH_2_CH(CH_3_)CH_2_CH_2_OH (**1c**)	66:0	85	3.5
4	*c*-C_5_H_9_NH_2_ (**1d**)	90:1	75	3
5	CH_2_=CHCH_2_NH_2_ (**1e**)	70:8	45	3
6	CH≡CCH_2_NH_2_ (**1f**)	93:0	75	3
7	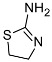 (**1g**)	10:0	90	68
8	PhNH_2_ (**1h**)	93:0	115	5
9	BnNH_2_ (**1i**)	80:10	95	3
10	*p*-C_2_H_5_-C_6_H_4_NH_2_ (**1j**)	89:0	90	4
11	*p*-CH_3_O-C_6_H_4_NH_2_ (**1k**)	98:0	95	3
12	NH_2_CH(CH_3_)CH_2_CH_3_ (**1l**)	95:2	70	4

^a^ Yields of pure isolated mono- and bis-adducts **3** and **4**; ^b^ Heating time.

**Table 2:** Yields and conditions for mono-addition of various primary multi-functional amines with methyl acrylate (**5**). 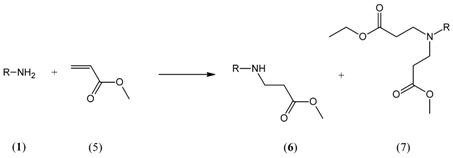

Entry	Amine (1)	Yield 6:7 (%) ^a^	Temperature (°C)	Time (Hours) ^b^
1	CH_2_=CHCH_2_NH_2_ (**1e**)	76 : 5	45	3
2	CH≡CCH_2_NH_2_ (**1f**)	84 : 8	75	3.5
3	*p*-CH_3_O -C_6_H_4_ CH_2_NH_2_ (**1m**)	91 : 0	90	4

^a^ Yields of pure isolated mono- and bis-adducts **6** and **7**; ^b^ Heating time.

**Table 3:** Yields and conditions for mono-addition of various amines with different Michael acceptors. 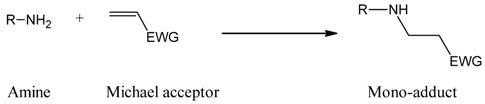

Entry	Acceptor	Amine (1)	Yield ^a^ (%) (Mono-Adduct)	Temperature (°C)/Time (h) ^b^
1	Acrylonitrile (**8**)	*n*-C_6_H_13_NH_2_ (**1b**)	98 (**9b**)	70/4
2	**8**	*c*-C_5_H_9_NH_2_ (**1d**)	92 (**9d**)	65/3.5
3	**8**	CH≡CCH_2_NH_2_ (**1f**)	83 (**9f**)	75/3.5
4	**8**	PhNH_2_ (**1h**)	90 (**9h**)	95/3
5	**8**	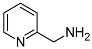 (**1n**)	92 (**9n**)	70/4
6	**8**	*p*-CH_3_OPhCH_2_NH_2_ (**1o**)	100 (**9o**)	90/4
7	**8**	CH(CH_3_)_2_NH_2_ (**1p**)	95 (**9p**)	−20/3
8	Acrylamide (**10**)	*n*-C_4_H_9_NH_2_ (**1a**)	95 (**11a**)	75/6
9	**10**	PhNH_2_ (**1h**)	90 (**11h**)	95/7
10	Methyl methacrylate (**12**)	*n*-C_4_H_9_NH_2_ (**1a**)	68 (**13a**)	75/5
11	**12**	*n*-C_6_H_13_NH_2_ (**1b**)	78 (**13b**)	90/5
12	**12**	*n*-C_5_H_11_NH_2_ (**1q**)	76 (**13q**)	90/5
13	Methyl *trans*-crotonate (**14**)	*n*-C_4_H_9_NH_2_ (**1a**)	71 (**15a**)	75/6
14	Methyl *trans*-cinnamate (**16**)	*n*-C_4_H_9_NH_2_ (**1a**)	43 (**17a**)	70/48

^a^ Yields of pure isolated mono-adducts; ^b^ Heating time.
